# Right-brain utilization in pharmacists' dispensing processes: an eye-tracking analysis of efficiency and safety using error-induction models

**DOI:** 10.1186/s40780-025-00443-4

**Published:** 2025-05-05

**Authors:** Toshikazu Tsuji, Kenichiro Nagata, Masayuki Tanaka, Shiori Iwane, Shigeru Hasebe, Yuto Nishiyama, Nana Yoshikawa, Hiroyuki Watanabe, Shigeru Ishida, Takeshi Hirota, Ichiro Ieiri, Mayako Uchida

**Affiliations:** 1 Department of Clinical Pharmacy, Setsunan University, Osaka, Japan; 2 Department of Pharmacy, Kyushu University Hospital, Fukuoka, Japan; 3 Department of Pharmacy, Fukuoka Tokushukai Hospital, Fukuoka, Japan; 4 Department of Pharmacology, School of Pharmaceutical Sciences, Fukuoka International University of Health and Welfare, Fukuoka, Japan

**Keywords:** Eye-tracking method, Thought process, Dispensing efficiency, Dispensing safety, Error-induction models, Right-brain utilization

## Abstract

**Background:**

Dispensing errors associated with “same-name drugs” and “similar-name drugs” are common, negatively affecting patients. Using two pairs of error-induction models, this study analyzed pharmacists' gaze movements while dispensing by an eye-tracking method to interpret their thought processes. Thus, we aimed to assess the efficiency and safety of dispensing processes by examining right-brain function using error-induction models.

**Methods:**

We created verification slides for display on a prescription monitor and three drug rack monitors. The prescription monitor displayed the dispensing information, including drug name, drug usage, location display, and total amount. A total of 180 drugs, including five target drugs, were displayed on the three-drug rack monitors. We measured total gaze points in the prescription area (Gaze 1), total gaze points in the drug rack area (Gaze 2), total vertical eye movements between the two areas (Passage), time required to dispense drugs (Time), and the error rate for each verification (Error). First, we defined two types of location display methods: “numeral combination” and “color/symbol combination”. Then, we established two pairs of error-induction models, F_1_-F_2_ (same-name drugs) and G_1_-G_2_ (similar-name drugs), to compare the differences between the two location display methods in the designated area.

**Results:**

Significant differences in gaze movements of pharmacists between the models F_1_-F_2_ were observed in Gaze 2, Passage, and Time (F_1_ > F_2_, *P* < 0.001, respectively), with similar results between models G_1_-G_2_ (G_1_ > G_2_, *P* < 0.001, respectively). Furthermore, the error rates in models F_1_ and F_2_ were 10.0% (11/110) and 6.4% (7/110), as well as 13.6% (15/110) and 5.5% (6/110) in models G_1_ and G_2_, respectively. A significant difference in error rates was observed between the models G_1_-G_2_ (G_1_ > G_2_,* P* = 0.020), but not between the models F_1_-F_2_ (*P* = 0.286).

**Conclusions:**

Incorporating visual information into prescription content not only performs a series of dispensing tasks more smoothly, but also reduces the error occurrences by pharmacists. In other words, leveraging right-brain utilization in dispensing processes has led to improvements in both efficiency and safety.

## Background

Safe medical care requires accurate drug dispensing. Pharmacists are responsible for dispensing a large volume of drugs accurately and quickly within a predetermined timeframe to support patients’ medical therapy. While many medical institutions have mechanized dispensing operations using one-dose package machines, pharmacists often face situations that require manual dispensing of drugs. Therefore, pharmacists need to develop more efficient dispensing methods and safeguard an environment that ensures accurate dispensing, even as mechanical support is introduced. Since the revision of content or procedures in dispensing work decreases the likelihood of errors, prompting ongoing efforts in various medical institutions to prevent such mistakes [1–12]. At Kyushu University Hospital, continuous efforts to prevent near misses and dispensing errors have kept the incidence rate of patients receiving incorrect drugs below 0.038% since 2006 [13–19]. However, given that human errors are inevitable, completely eliminating all dispensing errors remains a challenging goal. To improve the efficiency of dispensing work and decrease the likelihood of errors, pharmacists should understand their thought processes during complex and confusing dispensing situations. This understanding can help them develop measures to improve efficiency and safety in such circumstances.

Eye-tracking technology uses sensors that can detect and follow an individual’s eye movements in real time. In our previous studies, we used eye-tracking to clarify the basic confirmation process for target drugs in 12 pharmacists [20, 21]. We also analyzed the thought processes of 22 pharmacists in various dispensing environments [22]. Our findings revealed that pharmacists struggled with dispensing drugs located in the right area of drug racks. Additionally, we found that incorporating visual information into prescription content improved the efficiency of dispensing work. Specifically, the utilization of right-brain function in dispensing processes improved efficiency in handling drugs located in the right area of the drug racks. However, the safety of dispensing work associated with right-brain utilization remains unexplored in previous studies. In the present study, we first established two types of location display methods: “numeral combination” and “color/symbol combination”, to indicate the position of target drugs. We then carefully selected target drugs from two classifications: “same-name drugs” (F_1_-F_2_) and “similar-name drugs” (G_1_-G_2_). Furthermore, we prepared “error-induction models” to induce human errors intentionally by arranging each target drug between drugs of the same or similar classification. This approach allowed us to analyze the differences in efficiency and safety in dispensing work between each pair of error-induction models (F_1_-F_2_ and G_1_-G_2_). Although the specific details of left- and right-brain roles are not fully understood, it has been reported that the function of right-brain is closely linked to color recognition and processing [23]. Therefore, this study introduced visual information, such as colors or symbols, into the location display within prescription content. We analyzed both the efficiency and safety of dispensing work under complex and confusing conditions, which has not been thoroughly investigated in previous studies. Our goal was to demonstrate the effectiveness of using right-brain functions in dispensing work by analyzing the thought processes of pharmacists using error-induction models.

## Methods

### Gaze analysis using the eye-tracking system

Eye-tracking, which involves tracking the corneal reflection of infrared rays to verify gaze movements, is used in various fields, including medicine, psychology, and cognitive science [24–27]. In this study, we examined pharmacists’ gaze movements during the dispensing process using a wearable eye tracker (Tobii Pro Glasses 3, Tobii Technology K.K.). Gaze movement data were classified into two main categories: fixation (stagnation within a 20-pixel window for 100 ms or more) and saccade (quick movements of the eyeballs). Fixations and saccades were analyzed using motion videos recorded with dedicated software (Tobii Pro Lab Analyzer, Tobii Technology K.K.).

### Target persons and drugs

The inclusion criteria for pharmacists in this study were as follows: First, to ensure accurate eye movement measurements, pharmacists needed to be able to read dispensing information on large monitors without glasses (naked eyes or while using soft contact lenses). Second, pharmacists required more than 18 months of dispensing experience at Kyushu University Hospital to ensure a high level of verification quality. Finally, pharmacists had to agree to participate in the study.

The target drugs used in this study included five “same-name drugs” and five “similar-name drugs” dispensed at Kyushu University Hospital. “Same-name drugs” refer to drugs with identical names in katakana (character part) but differing in ingredient quantities (number part) as follows: Bisoprolol Fumarate 0.625 mg, 2.5 mg, and 5 mg; Efient® 2.5 mg, 3.75 mg, and OD 20 mg; Etizolam 0. 25 mg, 0.5 mg, and 1 mg; Alfacalcidol 0.25 µg, 0.5 µg, and 1 µg; and Rybelsus® 3 mg, 7 mg, and 14 mg. “Similar-name drugs” refer to drugs with more than three consecutive characters in katakana (character part) or more than two consecutive characters and the same ingredient quantity (character and number parts) as follows: Sitafloxacin 50 mg/Ciproxan 100 mg/Levofloxacin 250 mg, Diazepam 5 mg/Bromazepam 5 mg/Clotiazepam 5 mg, Candesartan 4 mg/Telmisartan 40 mg/Valsartan 80 mg, Rosuvastatin OD 2.5 mg/Atorvastatin 5 mg/Pravastatin Na 10 mg, and Alfacalcidol 0.5 µg/Eldecalcitol 0.5 µg/Rocaltrol® 0.5 µg. In this study, the five target “same-name drugs” were: Bisoprolol Fumarate 2.5, Efient® 3.75 mg, Etizolam 0.5 mg, Alfacalcidol 1 µg, and Rybelsus®3 mg. The five target “similar-name drugs” were: Ciproxan 100 mg, Bromazepam 5 mg, Telmisartan 40 mg, Atorvastatin 5 mg, and Eldecalcitol 0.5 µg.

### Verification slides

The slides used to verify dispensing in this study were generated using Microsoft PowerPoint® 2016. Each verification session consisted of one prescription slide and three drug rack slides. During each verification, five target drugs were dispensed. For each of the five target drugs, the dosage and administration were appropriate, and there were no drug interactions.

The prescription slide contained basic information at the top, including the patient's name, age (sex), body weight, height, and creatinine clearance value. In the center of the slide, the dispensing information was displayed, consisting of four items: (a) drug name, (b) drug usage, (c) location display, and (d) total amount. Each drug rack slide featured a grid layout with five rows and 10–14 columns, with each cell labeled with a drug name at the bottom. Three drug rack slides were prepared for each verification, with the five target drugs arranged at specified positions. Additionally, other “same-name” or “similar-name” drugs were arranged on both sides of each target drug to create a more complex and confusing dispensing environment, serving as “error induction models”. In total, approximately 180 drugs, including the five target drugs and 10 misleading drugs, were displayed across the three drug rack slides.

In this study, the indication method of (c) location display on the prescription slide was classified into two types: “numeral combination” and “color/symbol combination”. For the “numeral combination”, such as “2–1–10”, it indicated that the target drug was located on monitor- 2, in the first row from the top, and the tenth column from the left. Likewise, for the “color/symbol combination”, such as “  ”, it indicated that the target drug was located on monitor- 2, on the red line, and the third column from the right. In this method, five colors (red, yellow, green, blue, and black) were used, with a colored line shown above each row on the drug rack. Specifics of the dispensing information are presented in Table 1. In a series of studies, we conducted a total of seven pairs of dispensing verifications (Models A_1_-A_2_, B_1_-B_2_, C_1_-C_2_, D_1_-D_2_, E_1_-E_2_, F_1_-F_2_, and G_1_-G_2_), involving 14 prescription slides and 42 drug rack slides. The order of these verifications was randomized. For this study, we specifically analyzed data from two of the seven pairs of verifications (Models F_1_-F_2_ and G_1_-G_2_) to assess the efficiency and safety in dispensing work.

**Table 1:**
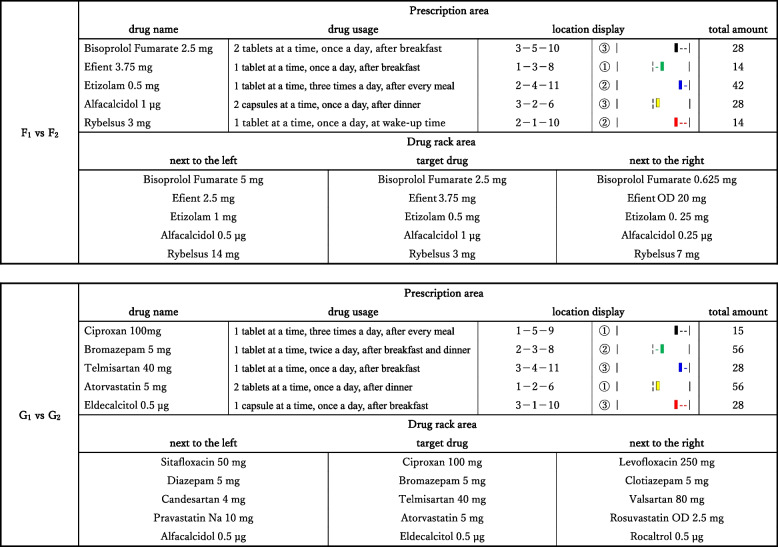
List of verification information

The object model and prescription information (drug name, drug usage, location display, and total amount) are displayed. Regarding location display, “2 - 1- 10” as an example of “numeral combination” means that the target drug is located on monitor 2, in the first row from the top, and the tenth column from the left. Likewise, “  ” as an example of “color/symbol combination” means that the drug is located on monitor 2, on the red line, and in the third column from the right

### Verification procedure

**Fig. 1:**
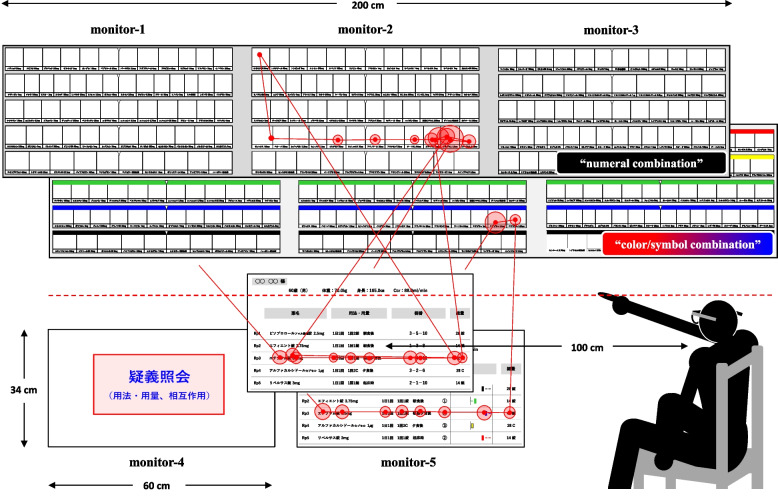
Outline of verification process using the eye-tracking method. Gaze movement data acquired by eye-tracking and analyzed using the Tobii Pro Lab Analyzer were mainly classified into two categories fixation (stagnation for a certain time) and saccade (quick movements of the eyeballs). We analyzed a series of dispensing processes by showing the prescription (34 cm × 60 cm) and drug rack (34 cm × 200 cm) areas. The red dotted line in the figure represents the boundary between the two areas. Wearing an eye tracker, the pharmacist was positioned on a chair 100 cm from monitor 5 and verified several pairs of dispensing information slides in random order

An outline of the verification task using the eye-tracking method is shown in Fig. 1. Five notebook computers were connected to 27-inch monitors (monitors 1–5) to operate the slides. The drug rack area (34 cm × 200 cm) was displayed on monitors 1, 2, and 3, positioned on the upper stage, while the prescription area (34 cm × 60 cm) was shown on monitor 5, located directly below monitor 2 on the lower stage. Monitor 4, used for prescription inquiries, was placed to the left of monitor 5. Wearing an eye tracker, the pharmacist was positioned 100 cm from monitor 5 to read the prescription slide. By showing the drug rack areas (on monitors 1, 2, and 3) and the prescription area (on monitor 5) simultaneously, we investigated the pharmacists’ gaze movements during the dispensing process. The Tobii Pro Lab Analyzer, which has recorded motion video, was used to assess various categories, including gazing point (center point in the circle), gazing time (size of the circle), and visual line movement (line between center points of circles).

To ensure the accuracy of the eye tracker, we calibrated it with each pharmacist before conducting the verification experiments. Pharmacists were allowed to practice with several training slides in advance to familiarize themselves with the verification process. The primary focus during the verification task was on maintaining smooth dispensing as usual. If a pharmacist noticed a mistake of selecting an incorrect drug during the dispensing process, they were allowed to correct it immediately. Furthermore, if the pharmacist identified an issue with the prescription content, they would point at monitor 4 for further inquiry. We analyzed the verification processes from the confirmation of dispensing information in the prescription area to the identification of five target spots in the drug rack area. The five major steps for dispensing verifications were as follows:

1. The pharmacist focused their gaze on a specific position.
2. When the pharmacist indicated the “Next” signal, the assistant switched to the corresponding prescription slide and three drug rack slides simultaneously.
3. The pharmacist read aloud the “total amount” of a target drug while pinpointing the target spot, repeating this process five times for each verification.
4. After the assistant pharmacist signaled “Next”, the assistant switched to a rest slide.
5. The sequence of verifications, involving 14 prescription slides and 42 drug rack slides, was repeated with voluntary breaks taken by the pharmacist as needed.

### Definition of the two paired models

**Fig. 2:**
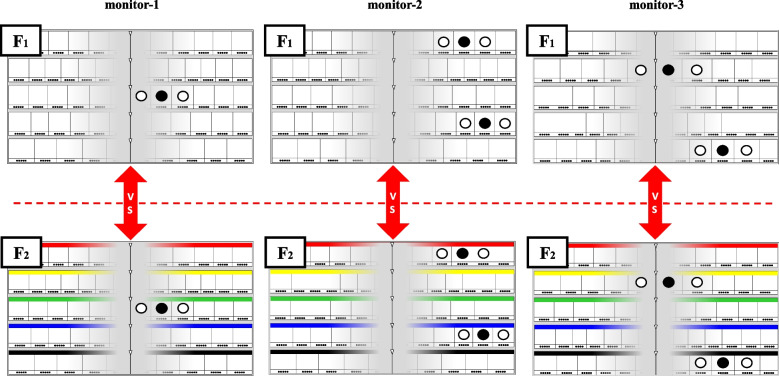
Arrangement of the five target drugs in error-induction models F_1_-F_2_ (same-name drugs). The location displays of the five target drugs in error-induction models F_1_-F_2_ (same-name drugs) are indicated using the “numeral combination (upper side)” and “color/symbol combination (lower side)”, respectively. These five target drugs and their locations in models F_1_-F_2_ are the same. The location spots of the five target drugs and both adjacent drugs having the same names in models F_1_-F_2_ are shown as black circles (●) and white circles (○), respectively

**Fig. 3:**
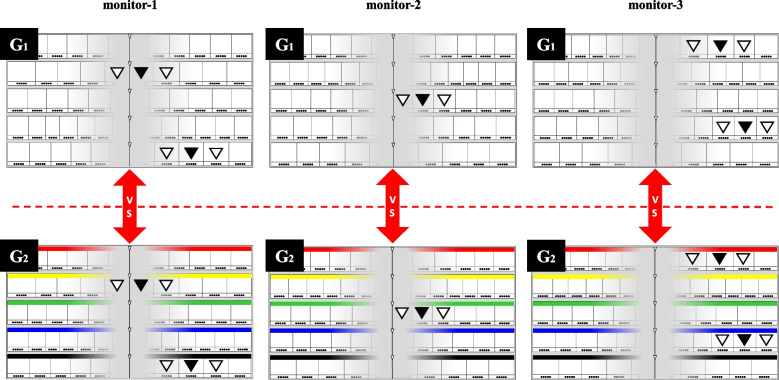
Arrangement of the five target drugs in error-induction models G_1_-G_2_ (similar-name drugs). The location displays of the five target drugs in error-induction models G_1_-G_2_ (similar-name drugs) are indicated using the “numeral combination (upper side)” and “color/symbol combination (lower side)”, respectively. These five target drugs and their locations in models G_1_-G_2_ are the same. The location spots of the five target drugs and both adjacent drugs having similar names in models G_1_-G_2_ are shown as black triangles (▼) and white triangles (▽), respectively

In a previous study, we established two pairs of basic models (A_1_-A_2_ and B_1_-B_2_) to compare the differences in gaze movements between the left and right areas, as well as three pairs of applied models (C_1_-C_2_, D_1_-D_2_, and E_1_-E_2_) to compare gaze movements between “numeral combination” and “color/symbol combination” in the left, center, and right areas. There were no dispensing errors generated in five pairs of models (A_1_-A_2_, B_1_-B_2_, C_1_-C_2_, D_1_-D_2_, and E_1_-E_2_) because they had no error-inductions. These models helped clarify differences in the efficiency of dispensing work based on drug locations or display types. In the present study, we developed two additional pairs of error-induction models (F_1_-F_2_ and G_1_-G_2_; Figs. 2 and 3) to compare gaze movements between the “numeral combination” and “color/symbol combination” specifically in the right area. These models were designed to intentionally induce errors, allowing us to analyze differences in both the efficiency and safety of the dispensing process.

The five target drugs and their locations remained consistent within each pair of models (F_1_-F_2_ or G_1_-G_2_), with the only difference being the location display method, either “numeral combination” or “color/symbol combination”. Below is a summary of the two pairs of error-induction models:

- Model F_1_-F_2_: This pair compares the “numeral combination” and “color/symbol combination” display methods in the right area using an error-induction model based on “same-name drugs”.
- Model G_1_-G_2_: This pair compares the “numeral combination” and “color/symbol combination” display methods in the right area using an error-induction model based on “similar-name drugs”.

### Verification items and classifications

To accurately dispense a target drug during the verification task, pharmacist follows these steps: First, the pharmacist should visually recognize four key items in the prescription area; (a) drug name, (b) drug usage, (c) location display, and (d) total amount. Second, the pharmacist should move their gaze upwards toward the drug rack area, which is considered as (e) vertical movement. Third, the pharmacist must identify the target drug by moving their gaze to the exact spot in drug rack area, and the number of gaze points required to reach the target spot is considered as (f) gaze arrival. Furthermore, reconfirming whether the identified target drug is correct was considered as (g) gaze addition (by moving the visual line down and up between the two areas). In this study, we measure the following parameters during the dispensing verification process: Gaze 1, the total number of gaze points in the prescription area; Gaze 2, the total number of gaze points in the drug rack area; Passage, the total number of vertical movements of visual lines across the boundary between the prescription and drug rack areas; and Time, the total time required to dispense the five target drugs.

We measured gaze movements of pharmacists and the time required for each verification, then analyzed the differences in them between the two location display methods “numeral combination” and “color/symbol combination”.

- Gaze 1: The total number of gaze points on the four items (a) drug name, (b) drug usage, (c) location display, and (d) total amount in the prescription area.
- Passage: The total number of (e) vertical movements between the prescription and the drug rack areas.
- Gaze 2: The total number of gaze points in the two items in the drug rack area (f) gaze arrival and (g) gaze addition.
- Time: The total time required to dispense five target drugs.

### Measurement of error occurrences

In the present study, we measured the number of errors occurring in each verification and analyzed the differences in error rates between the pairs of error-induction models (F_1_-F_2_ and G_1_-G_2_). “Error” in the dispensing process was defined as a situation where a pharmacist made a mistake of selecting an incorrect drug and could not correct it in the end. To determine error rates, we assessed each pharmacist’s performance, calculated the total number of errors, and then computed the average error rate for each model. This was done by dividing the total number of errors by the total number of target drugs (5 points × the number of target pharmacists).

### Data analysis

Using gaze category data (fixation and saccade), we analyzed Gaze 1, Gaze 2, Passage, Time, and Error (the average error rates in each model). Data are presented as the mean ± standard deviation. Differences between each pair of models in Gaze 1, Gaze 2, Passage, and Time were analyzed using the paired *t*-test. Differences in error rates were analyzed using McNemar's test. A *P-*value of < 0.05 was considered statistically significant, while *P-*values of < 0.01 and < 0.001 were considered highly significant. Statistical analyses were performed using JMP Pro 15 statistical software.

## Results

### Basic participating information and verification data

Participating in the study were 22 pharmacists (9 men and 13 women) with an average age of 30.1 ± 5.9 years. Among them, half (11/22; 4 men and 7 women) had less than 3 years of dispensing experience (average age, 25.3 ± 0.5 years), while the remaining half (5 men and 6 women) had more than 5 years of dispensing experience (average age, 34.9 ± 4.6 years).

### Comparison of gaze movements in the four classifications between two display methods

**Fig. 4:**
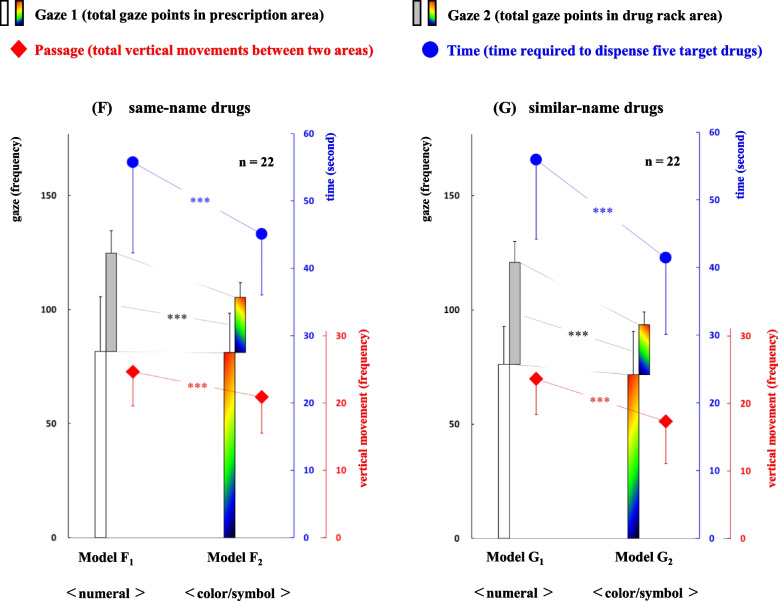
Comparison of gaze movement in the four classifications between two display methods in both error-induction models. Differences in gaze movements for Gaze 1, Gaze 2, Passage, and Time between two display types in both error-induction models F_1_-F_2_ (same-name drugs) and G_1_-G_2_ (similar-name drugs) are shown. ***P < 0.001 using the paired t-test

To clarify the difference in gaze movements of pharmacists between the two display methods “numeral combination” and “color/symbol combination” in the right area, we analyzed gaze movement data using two pairs of error-induction models. The data for the four classifications—Gaze 1, Gaze 2, Passage, and Time—are detailed below. Moreover, the relationships of the gaze movements between each pair of models, F_1_-F_2_ and G_1_-G_2,_ are shown in Fig. 4.

- Model F_1_: Gaze 1, 81.6 ± 24.1; Gaze 2, 43.0 ± 10.0; Passage, 24.6 ± 5.1; Time, 55.8 ± 13.5
- Model F_2_: Gaze 1, 81.2 ± 17.2; Gaze 2, 24.3 ± 6.4; Passage, 20.9 ± 5.4; Time, 45.1 ± 9.0
- Model G_1_: Gaze 1, 76.1 ± 16.6; Gaze 2, 44.5 ± 9.1; Passage, 23.6 ± 5.3; Time, 56.2 ± 11.8
- Model G_2_: Gaze 1, 71.6 ± 19.0; Gaze 2, 21.9 ± 5.5; Passage, 17.4 ± 6.3; Time, 41.6 ± 11.4

Significant differences between the models F_1_-F_2_ were observed in the three classifications: Gaze 2, Passage, and Time (*P* < 0.001, respectively). Likewise, significant differences between the models G_1_-G_2_ were observed in the three classifications: Gaze 2, Passage, and Time (*P* < 0.001, respectively).

### Comparison of gaze movements in the seven items per target drug between two display methods

**Fig. 5:**
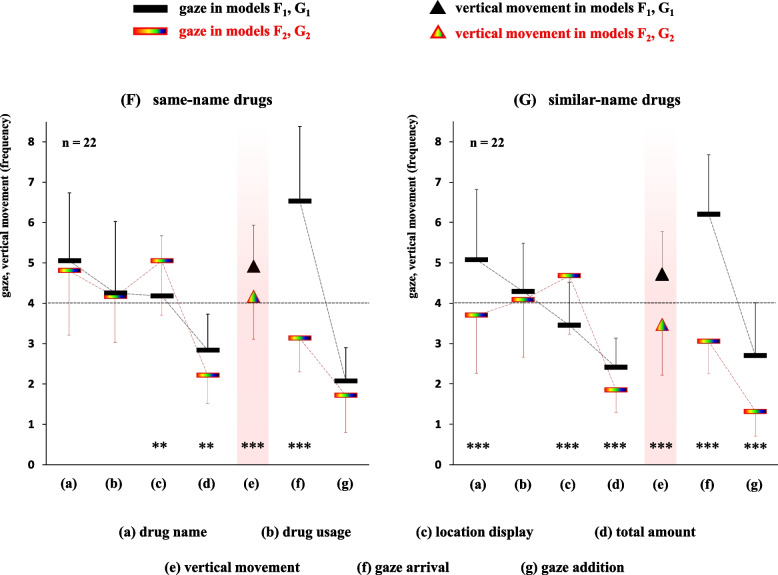
Comparison of gaze movements in the seven items per target drug between two display methods in both error-induction models. This figure depicts the differences in gaze movements for items (a) - (g) per target drug between two display types in both error-induction models. **P < 0.01, ***P < 0.001 using the paired t-test

To clarify the differences in gaze movements between “numeral combination” and “color/symbol combination”, we analyzed gaze movement data per target drug using two pairs of error-induction models. The gaze data for four items in the prescription area, (a) drug name, (b) drug usage, (c) location display, and (d) total amount, two items in the drug rack area (f) gaze arrival and (g) gaze addition, and the visual line data for (e) vertical movement between two areas were calculated. The relationships of the gaze movements between the models F_1_-F_2_ and G_1_-G_2_ are illustrated in Fig. 5.

- Model F_1_: (a), 5.1 ± 1.7; (b), 4.3 ± 1.8; (c), 4.2 ± 1.5; (d), 2.8 ± 0.9; (e), 4.9 ± 1.0; (f), 6.5 ± 1.9; (g), 2.1 ± 0.8
- Model F_2_: (a), 4.8 ± 1.6; (b), 4.2 ± 1.1; (c), 5.0 ± 1.3; (d), 2.2 ± 0.7; (e), 4.2 ± 1.1; (f), 3.1 ± 0.8; (g), 1.7 ± 0.9
- Model G_1_: (a), 5.1 ± 1.7; (b), 4.3 ± 1.2; (c), 3.5 ± 1.1; (d), 2.4 ± 0.7; (e), 4.7 ± 1.1; (f), 6.2 ± 1.5; (g), 2.7 ± 1.3
- Model G_2_: (a), 3.7 ± 1.4; (b), 4.1 ± 1.4; (c), 4.7 ± 1.4; (d), 1.9 ± 0.6; (e), 3.5 ± 1.3; (f), 3.1 ± 0.8; (g), 1.3 ± 0.6

Significant differences between the models F_1_-F_2_ were observed in the four items: (c) location display, (d) total amount, (e) vertical movement, and (f) gaze arrival (*P* < 0.01 or *P* < 0.001 for each item). Meanwhile, significant differences between the models G_1_-G_2_ were observed in the six items: (a) drug name, (c) location display, (d) total amount, (e) vertical movement, (f) gaze arrival, and (g) gaze addition (*P* < 0.001 for each item). Among them, the gaze frequency for (c) location display with the “color/symbol combination” was significantly higher compared to that of the “numeral combination” in both pairs of models (F_1_ < F_2_ and G_1_ < G_2_).

### Comparison of error rates between two display methods in both error-induction models

**Fig. 6:**
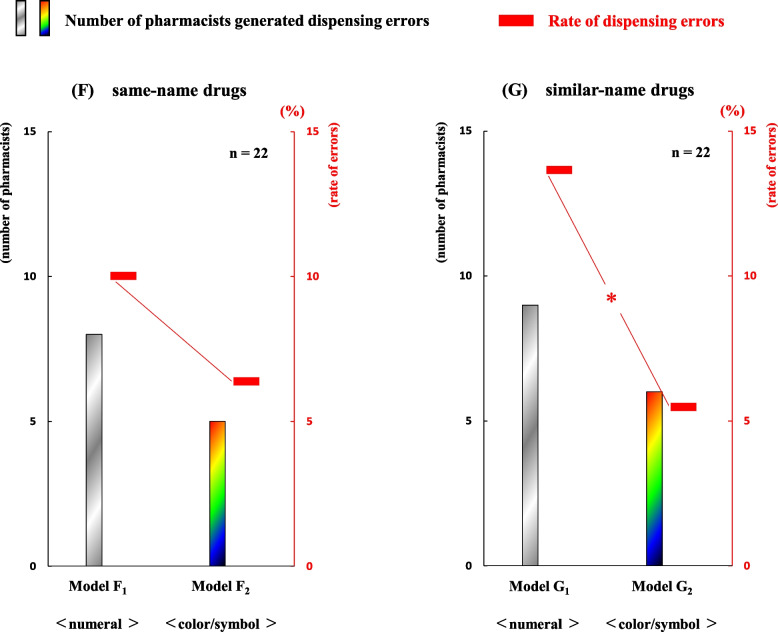
Comparison of error rates between two display types in both error-induction models. Differences in error rates between two display types in both error-induction models F_1_-F_2_ and G_1_-G_2_ are shown. *P < 0.05 using McNemar's test

To clarify the difference in error occurrences between the “numeral combination” and “color/symbol combination”, we analyzed the data from two pairs of error-induction models. The numbers of pharmacists with at least one dispensing error were eight and five in models F_1_-F_2_, and nine and six in models G_1_-G_2_, respectively. The numbers of dispensing errors were 11 and seven in models F_1_ and F_2_, and 15 and six in models G_1_ and G_2_, respectively. As a result, the error rates were 10.0% (11/110) and 6.4% (7/110) in models F_1_ and F_2_, as well as 13.6% (15/110) and 5.5% (6/110) in models G_1_ and G_2,_ respectively. No significant difference in error rates was observed between the models F_1_-F_2_ (*P* = 0.286). Meanwhile, a significant difference in error rates was observed between the models G_1_-G_2_ (with G_1_ > G_2_,* P* = 0.020). The relationships in the number of pharmacists with dispensing errors and the error rates between each pair of models F_1_-F_2_ and G_1_-G_2_ are shown in Fig. 6.

## Discussion

In the present study, we aimed to elucidate the thought processes of pharmacists in a complicated and confused dispensing environment, specifically focusing on the right-side location and error-induction situation, using an eye-tracking system. We analyzed differences in gaze movements of pharmacists between the location display methods “numeral combination” and “color/symbol combination” by setting up two pairs of error-induction models (same-name drugs; F_1_-F_2_, similar-name drugs; G_1_-G_2_). As a result, the method of information processing based on right-brain function enabled to identify directly the target point in prescription area without recognizing visually the both sides of it, which made it possible to decrease the additional checks in prescription/drug rack areas and the unnecessary vertical movements of visual lines between two areas. In this way, it was suggested that the avoidance of these vicious cycles in dispensing process led to reduce human errors, however, the details of why utilizing right-brain function would improve the efficiency and security of dispensing operations could not be clarified.

An important aspect of this study is that the only difference between the two location display methods “numeral combination” and “color/symbol combination” was pharmacist’s cognitive process or recognition method regarding drug’s positional information. First, the “numeral combinations” method (e.g., 2–4–11) functions as a translation code to convert numerical information into positional information, so that pharmacists must follow “L-shaped order” to locate the target drugs in the drug rack, moving from upper to lower and from left to right. When using “numeral combination”, especially in “error-induction models”, pharmacists need to think more deeply. The third number in the combination represents the horizontal drug position, making it challenging to remember and locate the target drugs in the right area of the rack, which is compounded by the limitation of human short-term memory. Research has shown that short-term memory typically holds 7 ± 2 items, but this can be reduced to 4 ± 1 items when information is complex or when there is interference [28–30]. This constraint underscores the difficulty pharmacists face in case of dealing with more intricate dispensing tasks involving numeral combinations, which requires deeper cognitive processing and can increase the risk of errors. Next, the “color/symbol combination” method (e.g.,  ) effectively uses colors to represent vertical direction and symbols to denote horizontal direction on the drug rack. Recognizing and processing colors is strongly related to right-brain function [23]. Therefore, when using the “color/symbol combination” method, pharmacists appear to identify the horizontal drug position with equal efficiency from both the left and right directions by perceiving it as part of the layout. For these reasons, incorporating visual information such as colors or symbols into prescription content enables pharmacists to identify the locations of target drugs more directly by utilizing right-brain function.

First, when comparing F1-F2 models (same-name drugs), significant differences in gaze movements of pharmacists during dispensing work were observed in Gaze 2, Passage, and Time, with F_1_ > F_2_ (*P* < 0.001 for each classification) across four classifications. Likewise, differences were noted between the G_1_-G_2_ models (similar-name drugs) for Gaze 2, Passage, and Time (G_1_ > G_2_, *P* < 0.001 for each classification; Fig. 4). These results suggest that a series of dispensing tasks using the “color/symbol combination” method was performed more smoothly than that using the “numeral combination” method, regardless of whether the target drugs were “same-name” or “similar-name”. In particular, the classification of Passage (vertical movements of visual lines between the prescription and the drug rack areas) may be an index of the flurried situation in dispensing process, since it means that pharmacists needed to reconfirm some kind of information across the upper and lower areas. However, no statistically significant differences were observed in Gaze 1 between the two pairs of models, leaving the impact of Gaze 1 on subsequent changes in Gaze 2, Passage, and Time unresolved.

Second, when examining the gaze movements per target drug across seven items (a)–(g) between the F_1_-F_2_ models, significant differences were found in three items: (d) total amount, (e) vertical movement, and (f) gaze arrival (F_1_ > F_2_, *P* < 0.01 or *P* < 0.001 for each item). On the other hand, significant differences between the G_1_-G_2_ models were observed in five items: (a) drug name, (d) total amount, (e) vertical movement, (f) gaze arrival, and (g) gaze addition (G_1_ > G_2_, *P* < 0.001 for each item; Fig. 5). An exception was observed in the gaze frequency for (c) location display, where the “color/symbol combination” method resulted in significantly higher gaze frequency compared to the “numeral combination” method for both pairs of models (F_1_ < F_2_ and G_1_ < G_2_, *P* < 0.01 or *P* < 0.001 for each item). This is likely because pharmacists could visually recognize the (c) location display at a glance in case of the “numeral combination” method (e.g., 2–1–10), apart from the subsequent translation from numerical information to positional information. In contrast, the “color/symbol combination” method (e.g.,  ) required pharmacists to divide it into two parts and check them separately, owing to the greater distance between two symbols in case of the right area. However, the increased gaze frequency for (c) location display in models F_2_ or G_2_ when using the “color/symbol combination” method seemed to have little effect on gaze movements in other items, as well as on Gaze 2, Passage, and Time, respectively (Figs. 4 and 5). In addition, it is notable that the gaze frequency for (d) total amount in model F_2_ was significantly lower than that in model F_1_, with a similar result between models G_1_ and G_2_. The item of (d) total amount was expressed almost entirely in numbers, suggesting that the incorporation of visual elements like colors or symbols had some effect on the memory storage of that numeral information. What is more notable is that significant differences in gaze movements of pharmacists between models G_1_ and G_2_ were observed in two items: (a) drug name and (g) gaze addition (G_1_ > G_2_, *P* < 0.001 for each item), but these differences were not present between models F_1_ and F_2_ (Fig. 5). This suggests that a difference emerged in the process of confirming (a) drug name and (g) gaze addition between the two pairs of models, though the specific factor responsible for these differences remains unclear based on the available data.

Third, while the error rate in model F_2_ was lower than that in model F_1_, no significant difference was observed between them. In contrast, the error rate in model G_2_ was significantly lower than that in model G_1_ (Fig. 6). Although the exact reason for the differing error rates between the two pairs of models (F_1_-F_2_ and G_1_-G_2_) is unclear, it is suggested that the significant decrease in gaze frequencies for both (a) drug name and (g) gaze addition in model G_2_ (similar-name drugs) contributed to the substantial reduction in error rate in for that model (Figs. 5 and 6). In other words, these results suggest that improving the efficiency of dispensing work leads to improved safety of it, which may be performed by incorporating visual elements like colors or symbols into prescription content.

Incidentally, considering the number of numeric items per target drug that needs to be memorized across four items (a), (b), (c), and (d) in the prescription, the totals for models F_1_, F_2_, G_1_, and G_2_ are five (a = 1, c = 3, d = 1), three (a = 1, c = 1, d = 1), four (a = 0, c = 3, d = 1), and two (a = 0, c = 1, d = 1), respectively. For models F_1_ and F_2_ (same-name drugs), prior memorization of the ingredient quantity (number part) of (a) drug name in the prescription area is essential. As a reason for that, pharmacists cannot distinguish the target drug from adjacent drugs with the same name in the drug rack area based solely on the memory of the katakana expression (character part), since they almost certainly recognize the plural ingredient quantities of “same name drugs” in their more than 18 months of dispensing experience. Furthermore, the three numbers in (c) location display (e.g., 2–1–10) for models F_1_ and G_1_ also function as a “translation code” required to convert the numerical information into position information. Meanwhile, prior memorization of numeric item in (a) drug name is not always necessary for models G_1_ and G_2_ (similar-name drugs) because the katakana expressions of the three adjacent drugs in the drug rack area are similar but distinct. In fact, the error rate in model G_2_, where only two numeric items needed to be memorized, was significantly lower than that in model G_1_. These results suggest that suppressing the number of numeric items requiring memory storage or conversion to minimum levels enabled to perform dispensing work without causing confusion under the complicated and confused dispensing environment, which may have led to a marked decrease in error rate. Therefore, minimizing the number of numeric items in the prescription content is crucial for ensuring both efficiency and safety in dispensing operations. To achieve this in practice, incorporating visual information like colors or symbols into the location display within the prescription content appears to be the most effective approach.

This study has some limitations. First, since the use of colors in the “color/symbol combination” display method may not be suitable for a pharmacist who has color vision deficiency, it is necessary to choose carefully the appropriate colors for him or her. Second, the range of available colors or symbols for dispensing information may be limited, and the display method used in this study may not apply to electronic medical charts across all medical environments. Furthermore, although the “color/symbol combination” method is effective for grid-type racks, its practical implementation in drawer-type racks may be challenging. However, to the best of our knowledge, this is the first study to clarify both the efficiency and safety of incorporating visual information such as colors and symbols into current dispensing practices and to evaluate the thought processes of over 20 pharmacists using an eye-tracking system. Therefore, the findings of this study may serve as a valuable reference for pharmacists in other facilities, as it demonstrates the benefits of integrating visual information based on right-brain function.

## Conclusions

We analyzed the differences in gaze movements of 22 pharmacists between the “numeral combination” and “color/symbol combination” location displays using an eye-tracking system. By employing two pairs of error-induction models (same-name drugs and similar-name drugs), we were able to elucidate pharmacists’ thought processes during complex and confusing dispensing tasks. Introducing visual information into prescription content not only performs a series of dispensing tasks more smoothly, but also reduces the error occurrences by pharmacists. In other words, utilization of right-brain processing in dispensing work can enhance both efficiency and safety in it.

## Data Availability

No datasets were generated or analysed during the current study.
